# Organ-on-a-chip models for development of cancer immunotherapies

**DOI:** 10.1007/s00262-023-03572-7

**Published:** 2023-11-03

**Authors:** M. Chernyavska, M. Masoudnia, T. Valerius, W. P. R. Verdurmen

**Affiliations:** 1 Department of Medical BioSciences, Radboud University Medical Center, Geert Grooteplein 28, 6525 GA Nijmegen, The Netherlands; 2 Division of Stem Cell Transplantation and Immunotherapy, Department of Medicine II, Christian-Albrechts-University, Christian-Albrechts-Platz 4, 24118 Kiel, Germany

**Keywords:** Organ-on-a-chip, Cancer immunotherapy, Advanced in vitro models, Microfluidics

## Abstract

Cancer immunotherapy has emerged as a promising approach in the treatment of diverse cancer types. However, the development of novel immunotherapeutic agents faces persistent challenges due to poor translation from preclinical to clinical stages. To address these challenges, the integration of microfluidic models in research efforts has recently gained traction, bridging the gap between in vitro and in vivo systems. This approach enables modeling of the complex human tumor microenvironment and interrogation of cancer-immune interactions. In this review, we analyze the current and potential applications of microfluidic tumor models in cancer immunotherapy development. We will first highlight current trends in the immunooncology landscape. Subsequently, we will discuss recent examples of microfluidic models applied to investigate mechanisms of immune-cancer interactions and for developing and screening cancer immunotherapies in vitro. First steps toward their validation for predicting human in vivo outcomes are discussed. Finally, promising opportunities that microfluidic tumor models offer are highlighted considering their advantages and current limitations, and we suggest possible next steps toward their implementation and integration into the immunooncology drug development process.

## Introduction

Cancer remains a leading cause of death globally despite the approval of many novel therapies, including some with long-lasting therapeutic benefits. In recent years, the paradigm-shifting approach of cancer immunotherapy has emerged as a transformative strategy, harnessing the body's own immune system to combat and even eradicate malignant tumors, thus revolutionizing the landscape of oncology treatment. In line with this, immunotherapy currently is the most rapidly growing field in cancer treatment, with the most prominent examples being divided into antibody-based and cell-based approaches. Monoclonal antibodies (mAbs), which are targeted against many cancer-associated markers (e.g., CD20, EGFR, HER-2, CD38) or checkpoint molecules, represent a large and rapidly growing class of cancer (immuno)therapies today, constituting of over 50% of more than 100 antibodies currently available for routine clinical use [1]. Progress in antibody engineering has allowed the generation of novel antibody-based molecules with desired effector functions or altered pharmacokinetics [2]. With the discovery of immune checkpoint-blocking antibodies, which typically block inhibitory receptors on T cells (such as CTLA-4, PD-1 and many others) recruitment of patients’ T cells to fight tumor cells has become an area of great attention [3]. Following the identification of classical T cell-recruiting checkpoint blockers also immune checkpoints on myeloid effector cells were identified, which negatively regulate antibody-dependent cellular phagocytosis (ADCP) by macrophages or antibody-dependent cell-mediated cytotoxicity (ADCC) by neutrophils. Here, the most prominent and clinically advanced example is the CD47/SIRPα axis, but antibodies against many other molecules are in the pipeline. Among the different antibody-based therapeutics, bispecific antibodies (BsAb) are currently a rapidly expanding class of molecules [4]. Many BsAb bind to tumor cell-expressed antigens and to CD3, thereby specifically recruiting and activating T cells. However, also Fc receptor-binding BsAb (e.g., against FcγRI) went into clinical trials early on and may re-emerge as interesting approach to recruit myeloid effector cells such as macrophages, neutrophils and dendritic cells (DCs) [5].

In addition to bispecific antibodies and checkpoint blockers, chimeric antigen receptor (CAR)-transduced T cells constitute the third pillar of approved T cell-recruiting immunotherapies. Typical CAR constructs are composed of an extracellular single chain antibody connected via a transmembrane region to intracellular activation (e.g., CD3ζ) and costimulatory domains (e.g., CD28 or 4-1BB) [6]. Depending on the signaling domains, CARs differ in the time course and intensity of T cell activation and their persistence in patients. Target antigens against which CARs have been approved so far are CD19 (four products) and BCMA (two products), but many others (e.g., CD22) are expected to follow soon. Typical clinical indications focus on hematological malignancies, while progress against solid tumors is hampered by toxicity issues [7].

In addition to CAR T cells, other engineered cell products are being developed and show promising results in early clinical studies. As an alternative to CAR-T, engineered T cell receptor (TCR)-expressing T cells (TCR-T) have been suggested for treatment of solid tumors. Recent clinical results of TCR-T therapies have been reviewed by Baulu et al*.* [8]. Natural killer (NK) cells represent an additional cell therapy source, potentially overcoming some of the CAR T-based limitations such as cytokine release syndrome or neurotoxicity. Currently, allogenic NK cell products are investigated in clinical trials and gave promising results in patients with acute myeloid leukemia and other hematological cancers (reviewed by Berrien-Elliott et al*.* [9]). Aside from antibody-based and cell-based approaches, vaccines against human papillomavirus and hepatitis B proved efficacious in preventing cervical and liver cancer, respectively [10]. Following up on the success of mRNA-based vaccines to prevent Covid-19 infections, also personalized therapeutic tumor vaccines hold promise, e.g., for treatment of pancreatic cancer [11] or melanoma [12]. Additionally, the use of engineered oncolytic viruses capable of attacking cancer cells and replicating in them without affecting healthy cells has expanded in recent years [13].

### Microfluidics in biomedicine

Despite the significant promise of cancer immunotherapies, the process of developing novel ones remains complex, time-consuming and costly, with low numbers of approvals, putting a price tag for a single oncology agent in a range of up to $4.5 billion according to market analysts [14, 15]. This prompts the need for “fast failure” solutions enabling early identification of ineffective drugs or their repurposing [16]. Poor translational value of preclinical models, which often fail to accurately recapitulate the human tumor microenvironment (TME), contributes to a high cost associated with cancer immunotherapy development. Whereas conventional two-dimensional cell culture models lack tissue complexity characteristics (morphology in 3D, extracellular matrix, gradients, physiological oxygen levels and vasculature), animal models come at low throughput and turnover, high costs, inability to dissect and control processes in the TME, and rodent models in particular have considerably different immune systems compared to the human immune system [17]. Therefore, microfluidic models offer a compelling alternative to traditional in vitro and animal models. These models present a unique advantage by accurately recapitulating complex physiological microenvironments, while also enabling dynamic and real-time analysis of cellular responses. Moreover, their inherent scalability, reduced sample requirements, and potential for high-throughput experimentation position microfluidic platforms as invaluable tools for advancing research in biomedicine.

Microfluidics emerged in the 1980s and is now a rapidly evolving field offering promising tools to bridge the gap between conventional in vitro models and in vivo models. In microfluidics fluids are manipulated at the micrometer scale with the help of miniaturized devices featuring interconnected channels, chambers and reservoirs with diameters typically ranging from tens to hundreds of micrometers. This allows for low Reynolds numbers and therefore laminar flow, giving more spatiotemporal control over the flow and molecule concentrations. The key interest in biomedical research is to accurately represent the physiology of tissues in so-called organs-on-chips, as it serves as a fundamental technology for creating physiologically relevant tissue models, ensuring better representation of the 3D tissue environment [18]. Depending on the purpose, microfluidic devices can be fabricated in various ways (e.g., molding, etching, soft lithography, 3D printing) and with different materials, such as polydimethylsiloxane (PDMS), silicon, glass, ceramic, and paper*.* Among materials used for manufacturing microfluidic chips, PMDS remains the most widely used material. PDMS is a low-cost, easily accessible material. Its optical transparency allows high-resolution imaging on-chip; flexibility enables production of valves and deformable elements; biocompatibility and gas permeability makes it an excellent material for biomedical applications [19, 20]. One of its major drawbacks, however, is hydrophobicity and absorption of small biomolecules, which has led to the development and concurrent use of alternative materials such as poly(methyl methacrylate) (PMMA). In the context of tumor-on-a-chip systems, the most widely used fabrication methods have been recently reviewed by Fang et al. [19].

The use of microfluidic devices also permits a multi-parameter readout, control of gradients (including oxygen control), compression and stretching of the tissue, application of sheer stress and interstitial flow, as well as integration of sensors measuring physical and chemical parameters (*e.g.*, electrical signals, pH, oxygen), reviewed by Ko et al*.* [20] and Palacio-Castañeda et al*.*[21]. The integration of engineering and cell culture techniques gave rise to microfluidic tumor models (tumors-on-chips) offering compartmentalized, more physiologically relevant and controllable representation of the TME, which are increasingly being used for immunotherapy development. This paper provides an overview of how the development of the key immunotherapy types (antibody- and cell-based therapies, cytokines, oncolytic viruses and cancer vaccines) benefit from using microfluidic platforms. It illustrates the promise, the limitations and future prospects of the development of cancer immunotherapies with microfluidic models, highlighting the conditions under which these models can facilitate bringing cancer immunotherapies from bench to bedside.

**Fig. 1:**
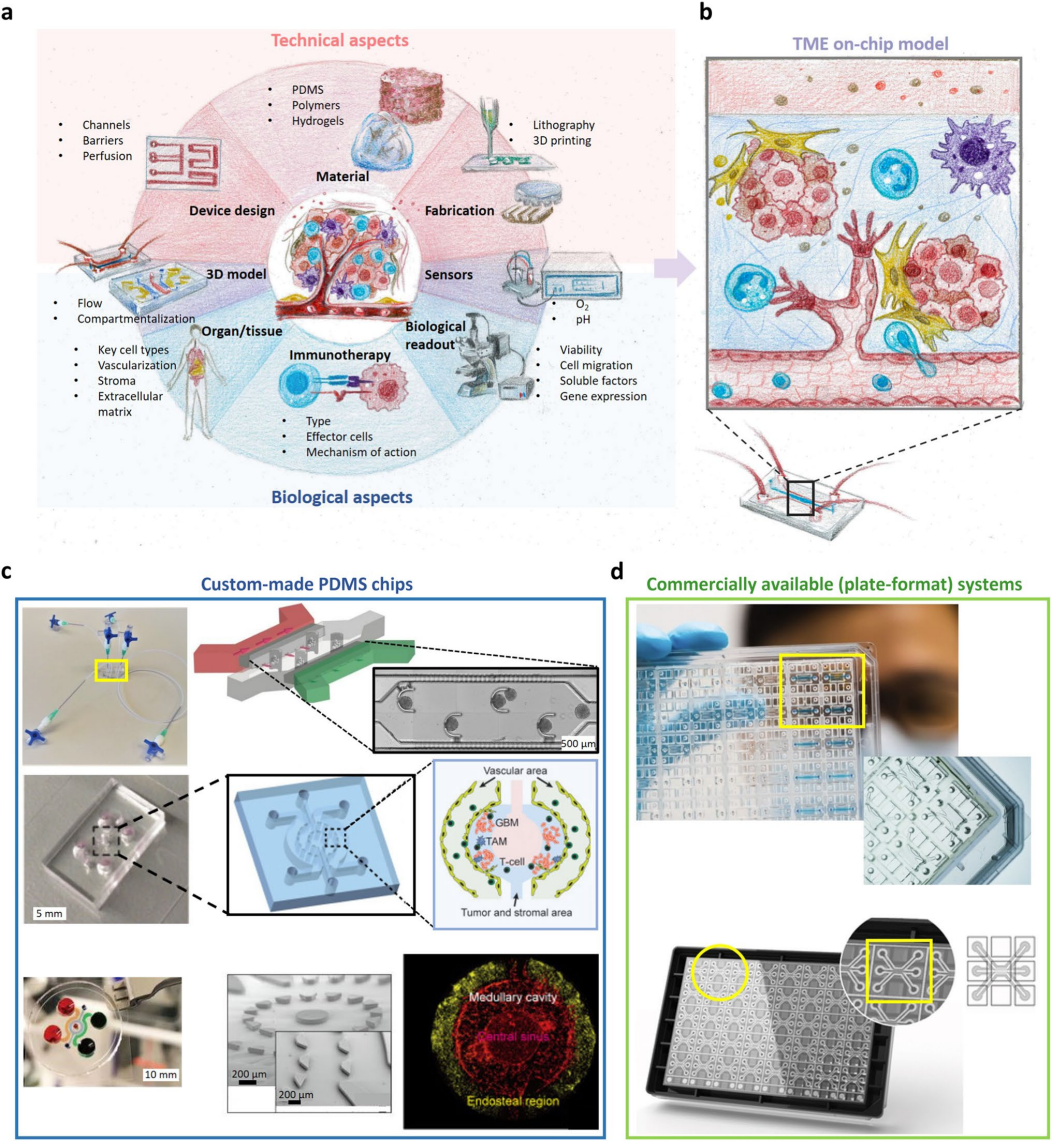
A schematic overview of the key technical and biological aspects of a microfluidic tumor model for cancer immunotherapy development and examples. (**a**) Various technical parameters (channel layout, fabrication material and technique) are considered depending on the biological features of the model (tissue, immunotherapy type and mechanism of action) and desired readout to evaluate immunotherapy. The 3D tissue model architecture on-chip and integration of sensors represent the intersection of biological and technical aspects of the models. (**b**) A resulting tumor microenvironment (TME) model (illustrated by a three-lane chip example) often includes various cells in a hydrogel perfused with a culture medium, optionally including a vascular tube or network and immune cells perfused or embedded in the hydrogel. (**c**) Examples of custom-made prototypical PDMS-based tumor-on-a-chip models with a lane design (top) or radial design (middle, lower). Photographs of the devices (left) are shown, as well as (schematic) channel arrangement (middle) and tissue model as a schematic or microphotograph (right); Top right images are reproduced from Chernyavska et al. [22] under a CC BY 4.0 license. Middle images on the right reproduced with permission from Cui et al. [23] under a CC BY 4.0 license. Lower images on the right reproduced with permission from Chen et al. [24] under a CC BY 4.0 license (**d**) Examples of commercially available plate-format systems featuring a three-lane design, by AIM Biotech (top) and MIMETAS (bottom). Top images from the right reproduced with permission from aimbiotech.com. Lower images on the right reproduced from Spijkers et al. [25] under a CC BY 4.0 license

Figure 1 summarizes the key technical and biological aspects of designing and fabricating microfluidic tumor models for cancer immunotherapy and illustrates several prototypical examples.

## Microfluidic tumor models in cancer immunotherapy development

Various microfluidic tumor models have been developed and applied for cancer immunotherapy development in recent years (Table 1). Most models contain parallel channels, including a tumor compartment with a hydrogel containing cancer cells or spheroids, and immune cells embedded in a hydrogel or perfused from a side channel. The specific choice of the design of the microfluidic model is typically guided by the research question being addressed, as the throughput, the dynamic characteristics (e.g., flow) and molecular readout possibilities vary widely between models, as detailed in Table 1. Below we will discuss the most recent applications of microfluidic models for development of the key types of cancer immunotherapies.

**Table 1:** Microfluidic tumor models used for cancer immunotherapy development

Type	Cell model	Device characteristics	Treatment	Readout on-chip	Key results	References
T cell, MAb, ICI	MDA-MB-231 Jurkat	Custom-made iHOC, 288 wells PDMS	Anti-PD1 MAb	Viability Tumor infiltration IL-2 concentration	Anti-PD1 MAb: Reverses tumor-mediated T cell inhibition Enhances IL-2 production Improves T cell infiltration and survival in the TME	[26]
T cell, MAb, ICI, Cytokine	MC38 CT26 (both ex vivo)	DAX-1, AIM Biotech (Bukit Merah, Singapore)	Anti-PD-1 mAb, IgG2a, anti-CD8a anti-IFN-γ, IL-6, TNF-α, LCL161	Viability IF Gene expression	Identified cells resistant to CD8 + T cell-mediated killing Dual PD-1 and Birc2/3 blockade reduces immunotherapy resistant cells and enhances tumor cell killing	[27]
T cell, MAb	GBM (patient-derived) hBMVECs TAMs Allogeneic CD8 + T cells	Three-channel radial design (outer: vessel, middle: tumor stroma, inner: medium), PDMS	Anti-PD-1 mAb, CSF-1R inhibitor (BLZ945)	Apoptosis TME composition Cytokines T cell activation Cell migration Gene expression DNA methylation	Patient-specific screening reveals distinct CD8 + T cell migration and interaction with TAM in different GBM subtypes CSF-1R and PD-1 dual blockade reverses immunosuppression elicited by M2-like TAMs	[23]
T cell, TCBs	Primary human alveolar epithelia Lung microvascular cells PBMCs HeLa Primary human intestinal organoids Colonic microvascular cells PBMCs	Emulate S-1 (Emulate, Boston, MA, USA) / porous membrane PDMS	Anti-FOLR1 TCB, anti-CEA TCB (high/low affinity)	IF FC Gene expression Viability Cytokines	FOLR-targeted TCB treatment did not cause lung toxicity On-chip responses are sensitive to the TCB affinity and mechanism of action Colon and Duodenum on-chips reveal target and mechanism-of-action-dependent toxicity differences	[29]
CAR T cells	anti-CD19 4-1BBζ-CAR T Reh B-ALL MSCs MSC-derived osteoblasts NHLFs Bone marrow mononuclear cells CD19 + K562 HUVECs	Glass-bonded PDMS chip, radial design (inner: central sinus; middle: medullary cavity; outer: endosteal region)	CAR-T cells	Gene expression IF T cell migration T cell activation T cell cytotoxicity T cell proliferation Cytokines	On-chip leukemia niche recapitulated physiological niche architecture and gene expression Relapse on-chip mirrored clinical relapse by antigen loss, expansion insufficiency, or patient T cell persistence CAR-T cells but not mock T cells: Completely eradicated leukemia blasts on-chip Induced systematic inflammation in niche Induced release of immunostimulatory and inflammatory cytokines	[24]
TCR engineering	HepG2 PBMC-derived T cells, expressing recombinant TCR	DAX-1, AIM Biotech	TCR-T cells, CRISPR-Cas9 for eTCR disruption, lentiviral delivery of HBV-specific rTCR	Viability T cell phenotype T cell cytokine response	TCR engineering led to a significant enrichment of HBV-specific populations rTCR expression increased along with enhanced cytokine production and improved killing of hepatoma cells on-chip	[28]
NK cell	HCT116 iPSC-derived cardiomyocytes NK cells	Modified Akura Flow MPS, InSphero (Schlieren, Switzerland)	NK cells	Apoptosis Cytokines NK cell activation NK cell migration Troponin I (soluble) Beating frequency	Safety and efficacy of NK treatment NK cell accumulated near shrinking tumor Non-responsive microtumors had fewer NK cells and larger size, influenced by soluble factors No cardiac defects, but decreased beating frequency in cytokine-rich environment	[32]
NK cell	HTLA-230 NB NK cells	MIVO device with a transwell insert (React4life, Genova, Italy))	NK cells	Flow NK cell migration and infiltration IF	NK cells actively extravasated into the tumor compartment with neuroblastoma cells under physiological flow conditions Preferential recruitment of CD16-negative NK cells resulting in cancer cell killing	[31]
NK cell, MAb, ICI	NK92-CD16 MiaPaCa-2 MCF-7 MDA-MB-231	330-micropillar chip platform, MBD Co. (Gwanggyo-ro, South Korea)	NK cells, trastuzumab, doxorubicin, paclitaxel, atezolizumab	Viability Apoptosis IF	Hypoxia mimicked on-chip Synergism by combining NK cells and trastuzumab/atezolizumab Lower EC_50_ values for paclitaxel indicate enhanced efficacy of mAb co-administration	[33]
NK cell, MAb, ICI	MCF-7 Breast carcinoma (patient) HUVECs NK-92	Two-layer, glass bonded PDMS chip, PDMS rod for channel formation	Atezolizumab, epacadostat	Cell migration Apoptosis Metabolism Gene expression	Initially cytotoxic NK cells became exhausted on-chip Limited NK cell cytotoxic capacity recovery even after removal of environmental stress Dual IDO-1 and PD-L-1 inhibition partially alleviated NK cell exhaustion and increased cancer cell death	[34]
MAb, ICI	EO771* (*ex vivo*)* ER + / PR + breast cancer (patient-derived) TNBC (patient-derived) RCC (patient-derived)	960 flow-unit (16 well) platform, PDMS	Anti-PD1, pembrolizumab	TME composition Viability Cytokines	On-chip responses of syngeneic EO771 cell lines predicted in vivo responses Differential therapeutic outcomes of PD-1 blockade in patient primary tumors on-chip	[35]
MAb, ICI	MDA-MB-468 HEK293 MDM (M1/M2)	OrganoPlate® 2-lane 96, Mimetas (Leiden, the Netherlands)	Anti-EGFR IgA, anti-EGFR IgG, SIRPα-Fc	Phagocytosis Cytokines Gene expression	Combination of anti-EGFR IgA and CD47 blocker: Induced synergistic anti-tumor activity Increased TNFα and IL-6 production Upregulated IL-1β, CD209, and CD163 in M2 macrophages	[36]
Targeting cytokines	MCF-7 CAFs MDA-MB-231	DAX-1, AIM Biotech	PFD	IF Cell migration Cytokines	PFD treatment of CAF: Reduced α-SMA expression Reduced CAFs and breast cancer cell migration Decreased invasiveness and immunosuppression of cancer cells by reducing CCL17 and TNF-β cytokine production	[37]
OV	A549 PBMCs	DAX-1, AIM Biotech	OVV	Apoptosis OVV infection Cell interaction	Synergistic killing of cancer cells using OVV and immune cells Significantly higher cancer cell killing for the combination treatment than OVV or PBMC alone, by enhancing cancer-immune cell interaction	[38]
Vaccines	T24 5637 MRC-5 HUVECs Macrophages	GelMA (bioprinted) in a casting with a membrane	BCG	Viability Proliferation Cytokines Growth factors	BCG treatment: Increased TNF-α, IL-12, IL-6, INF-y production Increased migration rates of macrophages in a dose-dependent manner	[39]
NK cell, RNA	Microglia U373MG GBM (patient-derived) NK92	Three-lane PDMS chip bonded on cover glass	miRNA-124 loaded in HEK293T-EVs	IF Cell migration Cell phenotype Gene expression Cytokines	EV-mediated miR-124 delivery: Suppressed GBM cell growth Inhibited M2, enhanced M1 glial cell polarization Reduced migration and morphology changes in both GBM cells and microglia Downregulated tumorigenesis factors Recruited NK cells to the TME Showed anti-tumor effects in patient-derived cell lines	[40]

BCG, Bacillus Calmette-Guérin; BLZ945, CSF-1R inhibitor; CEA, carcinoembryonic antigen; CRISPR-Cas9, clustered regularly interspaced short palindromic repeats-CRISPR-associated protein 9; CSF-1R, colony-stimulating factor 1 receptor; CSF-1, colony-stimulating factor 1; EC_50_, half maximal effective concentration; EGFR, epidermal growth factor receptor; ER+, estrogen receptor-positive; EV, extracellular vesicle; FC, flow cytometry; FOLR1, folate receptor 1; GBM, glioblastoma; HBV, hepatitis B virus; IF, immunofluorescence; IFN-γ, interferon gamma; IDO-1, indoleamine 2,3-dioxygenase 1; IL-2, interleukin-2; iPSC, induced pluripotent stem cell; mAb, monoclonal antibody; MDM M1/M2, monocyte-derived macrophages, M1-/M2-polarized; OV, oncolytic virus; OVV, oncolytic vaccinia virus; PD-1, programmed cell death protein 1; PD-L1, programmed death-ligand 1; PDMS, polydimethylsiloxane; PR+, progesterone receptor-positive; RCC, renal cell carcinoma; SIRPα-Fc, signal-regulatory protein alpha, Fc-fused; TCBs, T cell bispecific antibodies; TME, tumor microenvironment; TNF-α, tumor necrosis factor-alpha; TNBC, triple negative breast cancer. *Cell lines* 5637, human bladder carcinoma; A549, human lung adenocarcinoma; CAFs, cancer-associated fibroblasts; CT26, murine colorectal adenocarcinoma (ex vivo); EO771, murine mammary adenocarcinoma; GBM (patient), glioblastoma multiforme patient-derived; hBMVECs, human brain microvascular endothelial cells; HCT116, human colorectal carcinoma; HEK293, human embryonic kidney; HeLa, human cervical cancer; HTLA-230 NB, human neuroblastoma; iPSC-cardiomyocytes, induced pluripotent stem cell-derived cardiomyocytes; HUVEC, human umbilical vein endothelial cells; Jurkat, human T-cell leukemia; K562, human leukemia cell line; MCF-7, human breast adenocarcinoma; MDA-MB-231, MDA-MB-468, human breast adenocarcinoma; MC38, murine colorectal adenocarcinoma; MiaPaCa-2, human pancreatic adenocarcinoma; MRC-5, normal human lung fibroblasts; MSCs, human mesenchymal stem cells; NHLFs, normal human lung fibroblasts; NK-92, human natural killer cell line; PBMCs, peripheral blood mononuclear cells; RCC (patient), renal cell carcinoma patient-derived; Reh B-ALL, human acute B cell lymphoblastic leukemia cells; T24, human bladder cancer; TAMs, tumor-associated macrophages; U373MG, human glioblastoma

### T cell therapies

In the following section, we will highlight how a variety of T cell-based anti-cancer therapies was studied with microfluidic models, illustrating how these therapies influence dynamic immune cell interactions and therapeutic mechanisms in controlled microenvironments.

#### T cell-based mAb therapies

*Breast and colon cancer:* Jiang et al*.* [26] conducted a study investigating the effects of an anti-PD-1 antibody on T cells in a breast cancer model. The authors developed a custom-made immunotherapeutic high-throughput observation chamber (iHOC) consisting of 288 wells. The iHOC was produced by a combination of 3D printing, photolithography and PDMS replication and its optical transparency allows in situ observations of cellular processes with fluorescence and confocal microscopy. The researchers measured various parameters including MDA-MB-231 breast cancer spheroid and T cell viability, tumor infiltration, and IL-2 secretion. The concentration of IL-2 served as a marker of T cell activation as it is an established biomarker of activated T cells in vivo*,* promoting immune cell recruitment, growth and stimulation. The results showed that administration of the anti-PD1 monoclonal antibody reversed the PD-L1-mediated inhibition of T cells in a tumor spheroid size-dependent manner. Moreover, the anti-PD1 antibody increased IL-2 production, preventing T cell deactivation. Treatment with the anti-PD1 antibody also improved T cell infiltration and survival in the tumor microenvironment[26]. A similar approach was employed by Sehgal et al., who investigated the efficacy of an anti-PD1 antibody and targeted inflammatory cytokines in collagen-embedded murine colon cancer spheroids generated from ex vivo tissue [27]. In a commercial microfluidic device compatible with immunofluorescence staining, manual exchange of media and extraction of cellular material for RNA analysis, the authors utilized parallel analysis of viability, gene expression, and protein expression to identify a subpopulation of immunotherapy-resistant cells that avoided T cell-mediated killing. They also demonstrated the effectiveness of combining PD-1 blockade with Birc 2/3 (inhibitor of apoptosis members) antagonism to enhance cancer cell elimination [27].

*Glioblastoma*: The application of PD-1 blockade was also explored in the context of glioblastoma (GBM) by Cui et al*.* [23]. They designed a PDMS-based perfused chip that integrated patient-derived GBM cells, a functional vasculature, tumor-associated macrophages (TAMs), and allogeneic T cells. Through various analytical methods including cell viability, migration, and gene expression analysis, the authors investigated how different GBM subtypes affected T cell kinetics, infiltration, and interaction with tumor cells. They also demonstrated that dual blockade of PD-1 and CSF-1R, a survival-promoting receptor, could reverse the immunosuppressive effects of aggressive GBM. Moreover, the authors reported the immunohistochemistry and cytokine profile of their GBM on-chip model to be consistent with cytokine profiles of the patient GBM samples [23].

#### Cell therapies

*Leukemia:* A microfluidic model of a leukemia niche was developed and used to study CAR-T cell immunotherapy by Chen et al*.* [24]. The PDMS-based radial chip recapitulated the key components of the vascularized bone marrow stromal and immune niches, while a glass bottom allowed microscopic analysis. The authors then perfused anti-CD19 CAR-T cells and reported eradication of B-cell acute lymphoid leukemia (B-ALL) with potent induction of inflammation in the niche, which was not observed for mock T cells. The authors also modeled different responses to CAR-T cell therapy on-chip (remission, resistance, relapse) and highlighted that their model mirrored the clinical response and identified the factors for the potential therapeutic failure, e.g., relapse via insufficient expansion, patient T cell persistence, or surface antigen loss. Finally, the authors used their model to test available CAR-T cell products and demonstrated different responses in efficacy, suggesting the potential of use of microfluidic models as a “pre-clinical-trial-on-chip” tool for CAR-T cell therapy development [24].

*Liver cancer*: Another recent application of microfluidic systems in T cell-based therapies is exemplified by Preece et al*.,* who investigated the efficacy of T cell receptor (TCR)-engineered T cells to kill hepatoma tumor cells using a commercially available standard, three-lane chip model [28]. The authors disrupted endogenous TCR (eTCR) using CRISPR-Cas9 and introduced a Hepatitis B (HBV)-specific recombinant TCR (rTCR). They analyzed the phenotype and function of the engineered T cells both directly on-chip using microscopy and through cell recovery from on-chip culture. The study revealed increased expression of the rTCR compared to eTCR, along with enhanced cytokine production and killing of HBV antigen-expressing hepatoma cells on-chip [28].

#### Safety and alloreactivity of T cells

Kerns et al*.* conducted a study that validated their lung- and intestine-on-a-chip microfluidic models and demonstrated their application for the safety assessment of immunotherapies [29]. The authors developed a vascularized, perfused lung-on-a-chip, as well as colon and duodenum intestine chips with HeLa included as a cancer cell line, to examine cell toxicity in response to low- or high-affinity T cell bispecific antibodies (TCBs) targeting folate receptor (FOLR-1) and carcinoembryonic antigen (CEA), in healthy vs. tumor tissue. The microfluidic models predicted therapeutic windows for the TCBs to effectively eliminate cancer cells without inducing toxicity in healthy tissues. Importantly, the responses observed on-chip, but not in 2D culture, were sensitive to differences in affinity, target abundance, and mode of action of the TCBs. The same was observed in a cynomolgus monkey model, thereby demonstrating the usefulness of these models in predicting the therapeutic response in vivo [29].

There are concerns regarding the utilization of T cells in microfluidic tumor models for T cell-based therapy due to the risk of alloreactivity. However, it is important to note that alloreactive responses typically require a longer timeframe than what is typically employed in in vitro research, along with the involvement of other components of the adaptive immune system. Although in vitro studies with T cells, including microfluidic models, do not often report tumor cell killing without stimulation, the topic of alloreactivity is frequently overlooked. If necessary, mitigating alloreactivity can be achieved through measures such as matching HLA/MHC types between the tissue and donor T cells or utilizing patient explants and T cells from the same donor [30].

In summary, these examples underscore the versatility and potential of microfluidic models in unraveling the complexities of T cell-based cancer therapies, allowing for fine-tuned investigations into therapeutic efficacy, immune responses, and personalized treatments.

### NK cell therapies

#### NK cell-cancer interaction

The investigation of NK cell behavior and anti-cancer function within microfluidic tumor models offers valuable insights into their potential applications for cancer research and treatment. In studies by Nguyen et al. [32] and Marzagalli et al. [31] NK cell phenotype and function were interrogated in the context of colorectal adenocarcinoma and neuroblastoma models, respectively. In both cases, the authors adapted a commercial platform to enrich and analyze NK cells. The model reported by Nguyen et al. [32] also included a functional cardiac tissue to investigate tolerability, and the authors demonstrated selective NK cell-mediated tumor cell killing with varying response, and with no structural defects but decreased beating frequency of the cardiac tissue. Such a model serves as an example how tolerability can be evaluated in microfluidic tumor models alongside efficacy. The model reported by Marzagalli et al. [31] is focused on characterization of NK cell migration and extravasation. Therefore, it features a device where NK cells migrate against gravity and a fluid flow. The authors showed preferential recruitment of CD16-negative NK cells to the neuroblastoma spheroids and reported NK cell-mediated tumor cell death. These microfluidic tumor models thus provide useful tools for studying NK cell behavior, migration, and anti-cancer therapeutic efficacy, contributing to our understanding of their potential for future clinical applications.

#### NK cell-activating mAb therapies

While the previous studies focused on NK cell-cancer interactions and their functional aspects, there are additional investigations involving different treatments, primarily anti-PD1/anti-PD-L1 antibodies, similar to the mAb therapies mentioned earlier. The utilization of NK cell-activating mAb therapies, like anti-PD1 antibodies, constitutes a promising approach in cancer immunotherapy. By targeting the PD1/PD-L1 immune checkpoint, these therapies restore NK cell activity and enable enhanced tumor cell destruction, as highlighted in the studies described below.

As a first example, Gopal et al. [33] conducted a study using a high-throughput microfluidic device to explore the effects of combining trastuzumab (anti-HER2) and atezolizumab (anti-PD-L1) with doxorubicin and/or paclitaxel on NK cell-mediated killing of pancreatic or breast cancer cells. Their microfluidic system comprised 330 micropillar-microwell sandwich units for spheroid culture. The researchers demonstrated the induction of hypoxia in the TME within the spheroids and observed a reduction in the EC_50_ dose of small molecule chemotherapeutics when combined with NK cells and antibodies[33]. Similarly, Ayuso et al. [34] investigated the anti-cancer effects of atezolizumab and epacadostat (inhibitor of the potent immunosuppressor IDO-1) using a patient-derived breast cancer model in a custom-made PDMS device. Their chip included a channel patterned with endothelial cells to generate a functional vascular tube, along with a hydrogel that established a nutrient and metabolite gradient based on proximity to the tube. The authors analyzed NK cells recovered from the device for signs of exhaustion and reported the environmental stress impeded NK cell function, which could be partially restored by blocking PD-L1 and IDO-1.

In summary, these mAb-based studies further underscore the capacity of microfluidic platforms to dissect NK cell behaviors within intricate tumor microenvironments, contributing new insights into both the effectiveness of tumor cell killing and the broader implications for tissue interactions and tolerability.

### Other mAb therapies

While most studies investigating monoclonal antibody (mAb) efficacy focus on T cell or NK cell function, two studies that we discuss below explored the effectiveness of mAbs using different effector cell populations, namely a repertoire of patient-derived tissue-resident immune cells or monocyte-derived macrophages.

*Patient-derived immune cells:* Ao et al. conducted a study to assess the efficacy of murine and human anti-PD1 antibodies (including pembrolizumab) in mammary carcinoma models [33]. They utilized an ex vivo on-chip model and patient-derived material on a custom-designed platform with 960 flow units. The platform, made with PDMS, consisted of 16 channels with 60 wells each, enabling high-throughput analysis of tumor aggregates under 16 treatment conditions. The researchers evaluated cell viability, analyzed the inflammatory profile by measuring secreted factors and assessed tumor composition by examining cells recovered from the chips. This study included various tumor-resident immune cells derived from the patient's tumor microenvironment. Importantly, the on-chip response of a dissociated syngeneic murine mammary carcinoma (from E0771 orthotopic tumors) containing murine immune cells predicted the in vivo response in the respective mouse model. Moreover, primary tumor cells from dissociated patient tumors (breast cancer and renal cell carcinoma) loaded on-chip showed different response to PD-1 blockade. However, the authors did not have access to the respective clinical response data to correlate the on-chip predicted response with the clinical outcome. This study nevertheless provides a valuable validation of the on-chip response as a predictor of preclinical in vivo outcomes [35].

*Monocyte-derived macrophages:* In our own research, we focused on the phenotype and function of monocyte-derived macrophages as myeloid effector cells in an MDA-MB-468 breast cancer model [36]. We studied the combination treatment of anti-epidermal growth factor receptor (EGFR) IgA and a CD47 innate immune checkpoint blocker using a commercially available medium-throughput microfluidic platform compatible with high-resolution microscopy as well as extraction of media and cellular material for cytokine expression and RNA analysis, respectively [36]. Our findings demonstrated the synergistic activation of M2-macrophages in phagocytosing cancer cells by the combination, accompanied by the upregulation of pro-inflammatory cytokines TNFα and IL-6, as well as increased gene expression of IL-1β (M1 marker), CD209 and CD163 (M2 markers) [36]. Although this work was mostly focused on the role of the macrophage phenotype upon treatment with candidate cancer therapies, it also underscores the potential of other phagocytes to be employed as effector cells for cancer immunotherapy.

In essence, these two studies diverge from convention by illuminating mAb effectiveness through different effector cell types, reaffirming the dynamic capacity of microfluidic platforms in deciphering the complexities of cancer immunotherapies.

### Other immunotherapeutic approaches

Microfluidics additionally aids the study of emerging immunotherapies like cytokine targeting, oncolytic viruses, vaccines, and RNA-based agents. Cytokine targeting adjusts the immune response by modulating key signaling molecules, while oncolytic viruses selectively infect cancer cells for targeted destruction and immune activation. Cancer vaccines introduce tumor-related antigens to trigger immune responses. RNA-based methods, including vaccines, utilize RNA molecules to modify immune responses. Microfluidic platforms evaluate these strategies on cancer cells, fibroblasts and immune cells, highlighting their potential for enhancing cancer cell killing and immunostimulation in tumor models.

*Cytokine targeting and oncolytic virus:* In one study, Es et al*.* used a commercially available three-lane microfluidic chip to investigate the effects of pirfenidone (PFD), an antifibrotic agent with anti-inflammatory and antioxidant properties, on cancer-associated fibroblasts (CAFs) in a breast cancer model [37]. They found that PFD treatment reduced CAF and cancer cell migration and invasiveness by decreasing the production of immunosuppressive cytokines [37]. Mencattini et al. [38] utilized the same microfluidic platform to study oncolytic vaccinia virus (OVV) in a lung carcinoma model. Through a novel video-microscopy analysis, they observed enhanced immune cell recruitment and prolonged immune-cancer cell interactions in the presence of OVV, leading to increased cancer cell killing [38].

*Vaccine and RNA-based approaches*: In the context of bladder cancer immunotherapy, Kim et al. [39] created a 3D bladder model using bioprinting and investigated the effects of a Bacillus Calmette-Guèrin (BCG) vaccine. They found that BCG treatment increased the production of inflammatory cytokines and promoted macrophage migration, suggesting a potential immunostimulatory effect [39]. Hong et al. [40] used a PDMS-based microfluidic chip to study microRNA (miRNA)-based cancer immunotherapy in a GBM model. They demonstrated that extracellular vesicles (EVs) loaded with miRNA-124 (microglial regulator) effectively suppressed GBM growth and invasion, inhibited M2 microglial polarization, downregulated pro-tumorigenic factors, and promoted NK cell recruitment to the tumor microenvironment, and found it consistent with markers of these processes that were detected in a gene expression analysis of five patient-derived GBM cell lines[40].

## Conclusions and outlook

The field of microfluidic tumor models is rapidly expanding, with a growing number of applications in cancer immunotherapy. Compared to previous proof-of-concept studies with small molecules, the current state of the art in microfluidics enables integration of functional immune components to develop and study immunotherapies. Currently, such models are predominantly used for studying monoclonal antibodies, with many focusing on PD-1 blockade, aligning with the extensive research and widespread clinical approvals of PD-1 blockade as one of the most established clinically approved cancer immunotherapies at the moment [41]. As microfluidic models facilitate the modeling of interactions between immune effector cells and tumor cells, a growing number of on-chip studies is aimed at additional immunotherapeutic approaches utilizing effector functions of NK-, T-, and myeloid cells.

By enabling precise control over microenvironmental conditions, the presence or absence of specific cell types and advanced capabilities to follow in real-time on-chip molecular processes, the discussed studies have elucidated previously unexplored facets of immunotherapy, thereby generating novel insights into how they work at the molecular level and provide avenues for generating the next generation of immunotherapeutic approaches. A short-term implication is that the insights in T cell-based mAb therapies may yield novel strategies to better select patients that are likely to respond to specific existing immunotherapies, while in the long term these insights will facilitate the discovery and development of novel agents, which will expand beyond T cell-based mAb therapies and other existing therapies to fully novel immunotherapeutic approaches relying on activating a variety of immune cells, including but not limited to dendritic cells, NK cells, macrophages and neutrophils.

### Current limitations

Despite the advantages microfluidic models offer in the immunotherapy development, certain challenges remain. The fabrication process can be complex and often requires specialized expertise, limiting widespread adoption of microfluidic systems. Currently, the majority of end users are academic laboratories engaged in proof-of-concept studies. Standardization and reproducibility are additional challenges, as user-to-user variability can arise due to the intricate nature of the models and the inclusion of multiple compartments and parameters. Therefore, along with growing model complexity, there is a drive toward higher throughput, transitioning from custom home-made devices used in proof-of-concept studies to large-scale screening platforms compatible with automated loading, maintenance, and readout. Moreover, microfluidic model validation approaches face challenges. Although in vivo validation is deemed necessary, the scalability to human organs and the need for animal validation present practical constraints and may not fully reflect the complexity of the human immune system.

### Future perspectives

One key trend in microfluidic tumor models is the increasing complexity by implementation of more cell types, by including vascularization to simulate extravasation of therapeutic agents and immune cells and by establishing gradients on-chip. Furthermore, with a move toward coupling multiple tissues, the microfluidic field is evolving toward the development of multi-organs-on-chip systems [42].

To fully harness the potential of microfluidic tumor models in immunotherapy development, several necessary steps need to be taken. Standardization and further scaling are essential to reduce costs and enable automation and facilitate widespread adoption. Validation of microfluidic models, particularly their predictive value for immunotherapeutic responses (e.g., efficacy, specificity), remains a critical aspect, which can be achieved through either the use of animal tissues and comparison with the respective animal models, or through direct use of patient material on-chip correlated with clinical outcomes. Combining on-chip experimental platforms with in silico modeling approaches will enhance their predictive capabilities when used together. Additionally, addressing oxygen control within the system is crucial, with current efforts focusing on developing spheroids with a hypoxic core and generally improving control over oxygen and pH levels in tumors-on-chips, which are particularly relevant in cancer research [21]. Finally, shifting paradigms in academic translational research, integration of microfluidic tumor models into the regulatory network (such as the FDA Modernization Act endorsing animal-free alternatives including organs-on-chips) and in the discovery pipeline by industry players for toxicity and efficacy evaluation, will drive their broader utilization and impact in cancer immunotherapy research and development.
